# Supplementary material for: Disease‐modifying effects of ganglioside GM1 in Huntington's disease models
Source: EMBO Mol Med. 2017 Oct 9;9(11):1537–57. doi: 10.15252/emmm.201707763 (PMC5666311; doi:10.15252/emmm.201707763)
Supplement: Supplementary file 1 — Appendix [file EMMM-9-1537-s001.pdf]

APPENDIX

Disease-Modifying Effects Of Ganglioside GM1 In Huntington's Disease Models

Melanie Alpaugh[1,2], Danny Galleguillos[1,2], Juan Forero[2,3], Luis Carlos Morales[1], Sebastian W. Lackey[1], Preeti Kar[1], Alba Di Pardo[1¶], Andrew Holt[1], Bradley J. Kerr[2,4], Kathryn G. Todd[2,5], Glen B. Baker[2,5], Karim Fouad[2,3] and Simonetta Sipione[1,2,*]

Affiliations:
[1]Department of Pharmacology, University of Alberta, Edmonton, AB, T6G 2H7, Canada;
[2]Neuroscience and Mental Health Institute, University of Alberta, Edmonton, AB, T6G 2H7 Canada;
[3]Faculty of Rehabilitation Medicine, University of Alberta, Edmonton, AB, T6G 2G4, Canada;
[4]Department of Anesthesiology and Pain Medicine, University of Alberta, Edmonton, AB, T6G 2H7, Canada;
[5]Department of Psychiatry, University of Alberta, Edmonton, AB, T6G 2B7, Canada
[¶]Current address: Center for Neurogenetics and Rare Diseases, IRCCS Neuromed, Pozzilli, Italy

***Corresponding author:**

Simonetta Sipione, Department of Pharmacology, 9-21 Medical Sciences Building, University of Alberta, AB, T6G 2H7, Canada, ssipione@ualberta.ca

TABLE OF CONTENTS

- **Supplementary Materials and Methods**
- **Supplementary References**
- **Appendix Table S1.** *P* values for pairwise comparisons reported in the manuscript.
- **Appendix Table S2.** Statistical analysis of behavioural differences between Q7/140 and Q140/140 mice.
- **Appendix Fig. S1**. Animal models and experimental design.
- **Appendix Fig. S2.** Food consumption is not affected by GM1 in R6/2 mice.
- **Appendix Fig. S3**. Expression of major inflammatory cytokines is comparable between WT and R6/2 mice and is not significantly affected by treatment with GM1.
- **Appendix Fig. S4.** The behaviour of YAC128 mice in the elevated plus maze does not depend on mouse motor function and is affected by acute adinazolam treatment.
- **Appendix Fig. S5.** The behaviour of YAC128 mice in the forced swim test does not depend on mouse motor functions and is corrected by the anti-depressant imipramine.
- **Appendix Fig. S6.** Effect of short-term treatment with GM1 on the performance of YAC128 mice in the forced swim test.

SUPPLEMENTARY MATERIALS AND METHODS

Animal models. YAC128 mice overexpressing the human HD gene with 128 CAG repeats (Slow et al, 2003) were originally purchased from the Jackson Laboratories (Jackson Laboratories, Bar Harbor, ME, USA) and subsequently maintained on FVB genetic background in our animal facility at the University of Alberta. Q140 knock-in mice expressing a chimeric mouse/human exon 1 gene with approximately 140 CAG repeats (Menalled et al, 2003) were kindly donated by Cure HD Initiative (CHDI) and maintained on C57Bl/6J background in our animal facility. R6/2 mice overexpressing human HTT exon 1 (B6CBA-Tg (HDExon1)62Gpb/3J) (Mangiarini et al, 1996) were obtained at 5 weeks of age from the Jackson Laboratory (stock number 006494) and used for experiments between 6 and 12 weeks of age. All mice were maintained on a 14-10h light-dark cycle in a temperature and humidity-controlled room. All procedures involving animals were approved by the University of Alberta's Animal Care and Use Committee and were in accordance with the guidelines of the Canadian Council on Animal Care.

Inclusion/exclusion criteria. All mice were tested for motor performance prior to the beginning of experiments (baseline). Any WT animal that performed particularly poorly on motor tests, any HD mouse that had performance similar to WT mice, and any animal with body weight greater than 50 g was excluded from experiments. Mice were distributed across experimental groups based on body weight and motor performance at baseline, such that all groups of the same genotype averaged similar body weight and motor scores at baseline across four different motor tests. During experiments animals were excluded if overt abnormalities were observed in behavior or at dissection. Based on these criteria, a total of 4 mice were excluded, including 1 WT GM1 mouse that developed circling behavior, and 1 YAC CSF mouse due to the presence of

a mass in the brain at dissection and 2 more mice with blood clot in the brain or enlarged infusion site. Four R6/2 mice were excluded from biochemistry or neuropathology analysis as they died spontaneously and dissection of brain tissue could not be performed immediately at the time of death. Specific exclusion criteria were applied for individual tests: For the y-maze, mice that performed fewer than 5 entries were excluded. One animal met these criteria for exclusion. For the social approach test, any animal that did not show a preference for social interaction in the first part of the test was excluded from analysis of preference for social novelty in the second part of the test. Two mice per treatment group were excluded based on these criteria. For the open field, animals that jumped out of the open field during testing were excluded. One WT mouse treated with GM1 and one HD treated with CSF met these criteria for exclusion. Finally, mice were excluded from specific analysis if their performance was calculated to be a significant outlier (more than 1.96 standard deviations from the mean).

Intraventricular infusion of GM1. A microcannula (Alzet brain infusion kit 3) was stereotaxically implanted into the right lateral ventricle of anaesthetized mice and connected to an osmotic pump (Alzet mini-osmotic pump model 2004 for 28-day treatment, model 2006 for 42-day treatment) implanted subcutaneously on the back of the mouse. Because the infusion rate was different for the two pump models, the concentration of GM1 in the pump was adjusted to 3.6 mM (for model 2004) or 6 mM (for model 2006) to ensure equal GM1 dosing. GM1 (semi-synthetic) was provided by Seneb BioSciences Inc. (Holliston, MA). Control animals were infused with vehicle (artificial cerebro-spinal fluid, CSF, Harvard Apparatus).

Antibodies. Primary antibodies used were: rabbit anti-horse ferritin (1:400, Sigma), rabbit anti-glial fibrillary acidic protein (GFAP) (1:1000 for immunohistochemistry and 1:500 for immunobloting, Dako), rabbit anti-ionized calcium binding adapter (Iba1) (1:250 for

immunohistochemistry and 1:500 for immunoblotting, Wako), biotin-conjugated mouse anti-Neuronal Nuclei (NeuN) (MAB377B 1:250, Sigma), mouse anti-DARPP32 (1:2,000, BD Biosciences), rabbit anti-pThr34-Dopamine- and cAMP-regulated phosphoprotein (DARPP32) (1:2,000, Cell Signaling), mouse anti-alpha-tubulin (1:20,000, Sigma), rabbit anti-HTT N-18 (1:5,000, kindly provided by Dr. Truant, McMaster University), mouse anti-HTT MW8 (1:2,000, DSHB), mouse anti-HTT clone EM48 (1:1,000, Millipore), rabbit anti-HTT (PW0595, 1:2,000, Enzo) and mouse anti-HTT (MAB2166, 1:10,000, Millipore). Secondary antibodies used were donkey anti-rabbit Alexa488-conjugated (1:200, Invitrogen), biotinylated goat anti-rabbit (1:200, Vector labs BA-1000), goat anti-rabbit IRDye 680 and IRDye 800CW-conjugated and goat anti-mouse IRDye 680 and IRDye 800CW-conjugated (1:20,000 for immunoblotting and 1:40,000 filter trap assay, Li-Cor Biosciences).

Volumetric analysis. Brain volume was calculated based on the region between 1.98 mm to bregma and -2.3 mm to bregma. The striatum (bregma 1.98 mm to -2.3 mm), was manually traced from photomicrographs of NeuN-stained sections and using the "Mouse brain in stereotaxic coordinates" by Franklin and Paxinos for reference. The globus pallidus was not included in the analysis. The corpus callosum, (from bregma 0.02 mm up to -2.3 mm), was manually outlined from photomicrographs of sections stained with eriochrome cyanine, in order to calculate the area in each composite image. For the analysis of white matter tracts in the striatum, the latter was manually outlined and set as a region of interest (ROI). A threshold was set for the images based on slide background and then particle analysis was performed filtering by size ($>80\ \mu m^2$). All images were processed and analyzed using the NIH ImageJ software-based image processing package "Fiji". When multiple photographs were required for analysis, composite images were created using the "MosaicJ" plugin.

For calculation of Iba 1^+-, $GFAP^+$- and $ferritin^+$- areas, immunostained sections were microphotographed using a 20X objective to 8-bit images. A threshold was set based on section background and images were transformed to binary images. The area immunostained (% of total) was calculated by dividing the total area immune-positive by the area of the photomicrograph analyzed. For $NeuN^+$-cell count, size filtering was set to 80 pixels, to exclude non-specific background staining. Cell density of $NeuN^+$-cells in the striatum was calculated by dividing the number of cells counted by the number of images analyzed. Cells were counted from a minimum of 16 images per brain, ranging from 1.98 to 0.02 mm from bregma. The total number of neurons in this region of the striatum was estimated by multiplying the cell density by the previously calculated striatal volume. $Iba1^+$-cell density was calculated by dividing the total number of $Iba1^+$-cells in each image by the area of the image.

Eriochrome staining. White matter structures were stained with Eriochrome Cyanine R as previously described (Kiernan, 1984) with minor modifications. Briefly, slices were defrosted for 30 min and stained with Eriochrome Cyanine R solution (0.4% $FeCl_3$ w/v, 0.16% Eriochrome Cyanine R w/v and 0.4% H_2SO_4 v/v) for 1 h at RT. Sections were washed in ddH_2O for 1 min and then differentiated in 0.5% NH_4OH (v/v) for 10 sec. Slides were dehydrated in serial alcohol dilutions, followed by isopropanol and xylene prior to mounting with Permount (Fisher Scientific. Waltham, MA).

Body weight and food intake. Body weight was measured prior to the start of treatment and at day 21 after the beginning of treatment. Food intake was monitored daily from day 7 of treatment until day 21. Each mouse was provided with 10 g of chow diet/day and food was replaced daily, so that food consumption was not restricted. Food remaining in the cage was weighted at 1700 h.

Motor tests. R6/2 mice were tested between days 7-21 of treatment. Q140 mice were tested between days 28-42 of treatment (Fig. S1), as preliminary experiments in these mice showed only modest effects of GM1 on motor behavior prior to day 28 of treatment. For each specific test, all experimental groups were tested on the same day/s after the beginning of treatment.

Rotarod. Mice were tested in three consecutive trials of 3 min each, with 1 min rest in between trials, at fixed speed (12 RPM). The time spent on the rotarod in each of the three trials was averaged to give the overall latency to fall time for each mouse.

Narrow beam. Mice were placed at the extremity of a 100-cm-long wooden narrow beam (0.75 cm wide, suspended 30 cm above the floor) and allowed to traverse the beam from one extremity to the other three times, while being recorded with a video camera. Footfalls, body balance, and motor coordination were analyzed from videos using a footfall scoring system (Di Pardo et al, 2012).

Horizontal ladder. Mice were scored as they spontaneously walked along a horizontal ladder with irregular spacing between rungs. In each test session, mouse performance was evaluated using an established footfall scoring system (Metz & Whishaw, 2002).

Open field. Mice were placed in an open field apparatus (90 cm x 90 cm) for 5 min. The distance travelled by R6/2 mice in the last 3 min of a 5 min session was determined from video-recordings, by tracing the path travelled onto a grid. The distance travelled by Q140 mice in 5 min was analyzed using EthoVision XT tracking software.

Climbing test. The climbing test was performed according to (Hickey et al, 2008) with minor modifications. Briefly, mice were placed in a perforated wire container for 5 min and the number

of climbs (four paws on the side of the container) and rears (two paws on the side of the container) were counted. Only rearing behavior was analyzed as climbing behavior was extremely rare in all groups.

Kinematics analysis. Recordings from each animal were made 31 days after beginning of the treatment. Before recording, each animal was anaesthetized with isoflurane, and the location for the iliac crest on both hind legs was labeled with a spot of white paint. After full recovery from anesthesia, mice where placed into a custom-made Plexiglas walkway (length: 90 cm, width: 5 cm, height: 13 cm) with a mirror underneath that enabled monitoring lateral and ventral views of their free walking across the walkway. Two video recordings were captured for each walking direction using a high-speed video camera (200 frames per second) to monitor kinematic patterns. Video data were recorded and analyzed offline using a customized LabVIEW program. Footfall diagrams were obtained from identifying paw contact and paw lift on the ventral view, and these data were used to calculate stride duration (time between two consecutive paw contact with the ground), stance-to-stride ratio, and coupling between limbs during locomotion for each mouse (Leblond et al, 2003). Stick diagram representation of the hind limb based on the iliac crest marker and the paw captured from the lateral view were used to illustrate the step cycle of the mouse. The distance from the iliac crest marker to the ground was used to evaluate the ability of the mouse to support its weight.

Non-motor tests. YAC128 mice were tested between days 18-28 of treatment. Q140 mice were tested between days 25-38 of treatment. For each specific test, all experimental groups were tested on the same day after the beginning of treatment.

Elevated plus maze. Mice were placed in the centre of an elevated plus maze facing the open arm and left to freely explore for 5 min. Arm crosses into and out of open and closed arms, as well as time spent in each arm were recorded with a videocamera and scored. A subset of mice was injected intraperitoneally with adinazolam in saline solution (2.5 mg/kg, UpJohn Laboratories) 30 min prior to testing. Control animals received saline only.

Light-dark box test. The light-dark box test was performed as described in (Belzung et al, 1987) with minor modifications. Mice were placed in the dark half of a 700 mm x 300 mm black (dark) and white (light) box containing a tunnel joining the two halves. Mice were allowed to freely explore the box for 5 min. Time spent in each box, number of entries into each box, and latency to first entry were recorded.

Novelty-suppressed feeding test. The test was performed as in (Frye, 2007), with modifications. Briefly, 24 h prior to testing mice were introduced to the target food (diluted sweetened condensed milk) in their home cage. Animals were then food-deprived overnight (10-12 hours) to increase motivation to consume food. On the day of testing, mice were placed in a T-maze bated with diluted sweetened condensed milk at five places along the long arm. Mice were allowed to freely explore until they tasted the milk or for a maximum of 5 min. Time to approach and consume food was recorded.

Forced swim test. The forced swim test was performed as in (Porsolt et al, 1977). Mice were individually placed for 6 min in a 4L beaker (25 cm tall, 16 cm wide) filled with 2.6L of water pre-warmed to 23-25°C. The last four minutes of the test were scored using a time-sampling technique. Every five seconds mice were scored for swimming, climbing and immobility in the previous five seconds. Immobility was defined as no movement other than necessary to maintain

the nose above water. A subset of mice received an intraperitoneal injection of imipramine (10mg/kg in saline) or saline only (controls) 30 min prior to testing.

Simple swim test. The simple swim test was used to control for motor deficits potentially interfering with the forced swim test (Pouladi et al, 2009). One day prior to testing, mice were trained to swim in a rectangular swimming chamber (90 cm long x 7 cm wide; water depth = 9 cm; with 6x7 cm platform at one end) and to reach a platform at one end of the swimming chamber in three consecutive trials separated by 5 min intervals. On the day of testing, swimming speed was calculated by averaging the time each mouse took to swim the length of the pool to the platform in 7 consecutive trials, after excluding from the count the best and worst trial for each animal (Pouladi et al, 2009).

Open pool test. The open pool test was performed 1-2 days after the forced swim test. Mice were placed in a pool (102 cm diameter, 21 cm high) filled with water at a temperature of 23-25ºC for 6 minutes. Swim activity in the last 4 minutes was measured as described above for the forced swim test.

Nest building. Nesting 9ehavior was assessed as described in (Deacon, 2006) with modifications. Individually-housed mice were given a piece of paper towel and no other enrichment devices in their home cage. Percentage of paper towel shredded and height of the nest were scored according to a five point scale (Deacon, 2006) after 10 days for YAC128 and 2 days for Q140 mice.

Open field habituation and spontaneous defecation. Mice were placed in an open field apparatus (90 cm x 90cm) and filmed during 30 min sessions on two consecutive days as they explored the environment. Distance travelled in each 5 min interval was measured using EthoVision XT

tracking software. Intrasession habituation was expressed as activity change ratio and calculated using the formula: *distance travelled in the last 5 min/sum of distance travelled in the first and in the last 5 min.* Intersession habituation was also expressed as activity change ratio between first session on day 1 and second session on day 2, and calculated as: *total distance travelled on day 2/sum of distance travelled on day 1 and day 2* (Bolivar et al, 2002; Cook et al, 2002). At the end of each 30 min session, the number of fecal pellets dropped by each mouse was counted.

<u>Social approach test</u>. The social approach test was performed as described in (Ey et al, 2012). The test consisted of three 5 min sessions: 1) habituation, 2) social interaction and 3) social novelty. In session 1 each mouse was placed in the central compartment of an empty plexiglass box divided into three compartments (left, center and right) and left to explore for 5 min. In session 2 (social interaction), a second unfamiliar mouse of the same age, sex and genotype (stranger 1) as the test mouse was placed inside a metal mesh container located in the left or right compartment of the box in an alternating pattern. In session 3 (social novelty) a third mouse of the same sex and genotype (stranger 2) was placed in the opposite compartment of the box, inside a metal mesh container. Time spent by the test mouse in each compartment in each session was recorded.

<u>Y-maze</u>. Mice were placed in a Y-maze with three identical arms (30.5 cm long x 5 cm wide, with 16 cm-high walls) and left to explore the maze for 5 min. Time of first entry and arm entries were recorded. An entry was defined as the front half of the mouse body being inside an arm. Percent correct alternations was calculated by giving one point for every correct alternation and applying the formula: *number of correct alternations/(total number of arm entries – 2)* (Hughes, 2004). Animals that made fewer than five entries were excluded from the analysis (Swonger &

Rech, 1972). In our experiments only one mouse out of a total of forty-four met the criteria for exclusion (Swonger & Rech, 1972).

SUPPLEMENTARY REFERENCES

Belzung C, Misslin R, Vogel E, Dodd RH, Chapouthier G (1987) Anxiogenic effects of methyl-beta-carboline-3-carboxylate in a light/dark choice situation. Pharmacology, biochemistry, and behavior 28**:** 29-33

Bolivar VJ, Scott Ganus J, Messer A (2002) The development of behavioral abnormalities in the motor neuron degeneration (mnd) mouse. Brain Res 937**:** 74-82

Cook MN, Bolivar VJ, McFadyen MP, Flaherty L (2002) Behavioral differences among 129 substrains: implications for knockout and transgenic mice. Behav Neurosci 116**:** 600-611

Deacon RM (2006) Assessing nest building in mice. Nature protocols 1**:** 1117-1119

Di Pardo A, Maglione V, Alpaugh M, Horkey M, Atwal RS, Sassone J, Ciammola A, Steffan JS, Fouad K, Truant R, Sipione S (2012) Ganglioside GM1 induces phosphorylation of mutant huntingtin and restores normal motor behavior in Huntington disease mice. Proc Natl Acad Sci U S A 109**:** 3528-3533

Ey E, Yang M, Katz AM, Woldeyohannes L, Silverman JL, Leblond CS, Faure P, Torquet N, Le Sourd AM, Bourgeron T, Crawley JN (2012) Absence of deficits in social behaviors and ultrasonic vocalizations in later generations of mice lacking neuroligin4. Genes, brain, and behavior

Frye AAWaCA (2007) The use of the elevated plus maze as an assay of anxiety-related behavior in rodents. Nature protocols**:** 322-328

Hickey MA, Kosmalska A, Enayati J, Cohen R, Zeitlin S, Levine MS, Chesselet MF (2008) Extensive early motor and non-motor behavioral deficits are followed by striatal neuronal loss in knock-in Huntington's disease mice. Neuroscience 157**:** 280-295

Hughes R (2004) The value of spontaeous alternation behavior (SAB) as a test of retention in pharmacological investigations of memory. Neuroscience and Biobehavioral Reviews 497-505

Kiernan JA (1984) Chromoxane cyanine R. II. Staining of animal tissues by the dye and its iron complexes. Journal of microscopy 134**:** 25-39

Leblond H, L'Esperance M, Orsal D, Rossignol S (2003) Treadmill locomotion in the intact and spinal mouse. J Neurosci 23**:** 11411-11419

Mangiarini L, Sathasivam K, Seller M, Cozens B, Harper A, Hetherington C, Lawton M, Trottier Y, Lehrach H, Davies SW, Bates GP (1996) Exon 1 of the HD gene with an expanded CAG repeat is sufficient to cause a progressive neurological phenotype in transgenic mice. Cell 87**:** 493-506

Menalled LB, Sison JD, Dragatsis I, Zeitlin S, Chesselet MF (2003) Time course of early motor and neuropathological anomalies in a knock-in mouse model of Huntington's disease with 140 CAG repeats. The Journal of comparative neurology 465**:** 11-26

Metz GA, Whishaw IQ (2002) Cortical and subcortical lesions impair skilled walking in the ladder rung walking test: a new task to evaluate fore- and hindlimb stepping, placing, and co-ordination. J Neurosci Methods 115**:** 169-179

Porsolt RD, Le Pichon M, Jalfre M (1977) Depression: a new animal model sensitive to antidepressant treatments. Nature 266**:** 730-732

Pouladi MA, Graham RK, Joshi P, Lu G, Deng Y, Wu N-P, Figueroa BE, Metzler MA, Slow EJ, Raymond L, Friedlander R, Levine MS, Leavitt BR, Hayden MR (2009) Diffeential susceptivility to excitotoxic stress in YAC128 mouse models of HD between initiation and progression of disease. Journal of Neuroscience**:** 2193-2204

Slow EJ, van Raamsdonk J, Rogers D, Coleman SH, Graham RK, Deng Y, Oh R, Bissada N, Hossain SM, Yang YZ, Li XJ, Simpson EM, Gutekunst CA, Leavitt BR, Hayden MR (2003) Selective striatal neuronal loss in a YAC128 mouse model of Huntington disease. Human molecular genetics 12**:** 1555-1567

Swonger AK, Rech RH (1972) Serotonergic and cholinergic involvement in habituation of activity and spontaneous alternation of rats in a Y maze. Journal of comparative and physiological psychology 81**:** 509-522

SUPPLEMENTARY FIGURES AND TABLE

Appendix Table S1 – *P* values for pairwise comparisons reported in the manuscript

Figure	Panel	Groups compared	*p*-value
Figure 1	A	WT CSF vs HD CSF<br>WT GM1 vs HD GM1<br>HD CSF vs HD GM1	$p<0.0001$<br>$p<0.0001$<br>$p=0.008$
	B	WT CSF vs HD CSF<br>WT GM1 vs HD GM1<br>HD CSF vs HD GM1	$p=0.0057$<br>$p=0.6267$<br>$p=0.0320$
	C	WT CSF vs HD CSF<br>WT GM1 vs HD GM1<br>HD CSF vs HD GM1<br>WT CSF vs WT GM1	$p<0.0001$<br>$p=0.0003$<br>$p=0.0092$<br>$p=0.0355$
	D	HD 6 weeks vs HD 8 weeks<br>HD 6 weeks vs HD GM1<br>HD 6 weeks vs HD CSF<br>HD 8 weeks vs HD CSF<br>HD 8 weeks vs HD GM1<br>HD CSF vs HD GM1	$p=0.0212$<br>$p=0.0212$<br>$p<0.0001$<br>$p=0.0088$<br>$p=0.999$<br>$p=0.0077$
	E	WT CSF vs HD CSF<br>WT GM1 vs HD GM1<br>HD CSF vs HD GM1	$p=0.0096$<br>$p=0.9553$<br>$p=0.0097$
	F	WT CSF vs HD CSF<br>WT GM1 vs HD GM1<br>HD CSF vs HD GM1	$p<0.0001$<br>$p=0.0005$<br>$p=0.0473$
	G	WT CSF vs HD CSF<br>WT GM1 vs HD GM1<br>HD CSF vs HD GM1	$p<0.0001$<br>$p=0.2930$<br>$p=0.0019$
	H	WT CSF vs HD CSF<br>WT GM1 vs HD GM1<br>HD CSF vs HD GM1	$p<0.0001$<br>$p=0.1371$<br>$p=0.0497$
	I	HD CSF vs HD GM1	$p=0.10$
Figure 2	B	WT vs R6/2 Cortex	$p=0.049$
	C	WT CSF vs HD CSF Cortex<br>R6/2 CSF vs R6/2 GM1	$p=0.0019$<br>$p=0.028$
	E	WT CSF vs R6/2 CSF Striatum<br>WT CSF vs WT GM1 Striatum<br>WT CSF vs R6/2 GM1 Cortex	$p=0.0326$<br>$p=0.0335$<br>$p=0.0350$
	F	WT CSF vs WT GM1 Striatum<br>R6/2 CSF vs R6/2 GM1 Striatum<br>WT CSF vs HD CSF Cortex	$p=0.0415$<br>$p=0.0255$<br>$p=0.0011$
	G	WT CSF vs R6/2 CSF<br>R6/2 CSF vs R6/2 GM1	$p=0.0145$<br>$p=0.0194$
Figure 3	A	Q140 CSF vs Q140 GM1 mHTT	$p=0.0231$

Appendix Table S1 – *P* values for pairwise comparisons reported in the manuscript (Cont.)

Figure	Panel	Groups compared	*p*-value
Figure 3	C	Q140 CSF vs Q140 GM1	$p=0.005$
	D	R6/2 CSF vs R6/2 GM1	$p=0.0317$
Figure 4	A	R6/2 CSF vs R6/2 GM1 day 7<br>R6/2 CSF vs R6/2 GM1 day 11<br>R6/2 CSF vs R6/2 GM1 day 15<br>R6/2 CSF vs R6/2 GM1 day 21	$p<0.01$<br>$p<0.01$<br>$p<0.01$<br>$p<0.01$
	B	Q7/7 CSF vs Q140 CSF<br>Q7/7 CSF vs Q140 GM1<br>Q140 CSF vs Q140 GM1	$p<0.0001$<br>$p=0.016$<br>$p=0.0014$
	C	R6/2 CSF vs R6/2 GM1 day 7<br>R6/2 CSF vs R6/2 GM1 day 11<br>R6/2 CSF vs R6/2 GM1 day 15<br>R6/2 CSF vs R6/2 GM1 day 21	$p<0.05$<br>$p<0.01$<br>$p<0.001$<br>$p<0.001$
	D	Q7/7 CSF vs Q140 CSF<br>Q140 CSF vs Q140 GM1	$p<0.0001$<br>$p=0.0078$
	E	Q7/7 CSF vs Q140 CSF<br>Q140 CSF vs Q140 GM1	$p=0.0008$<br>$p=0.0005$
	F	Q7/7 CSF vs Q140 CSF<br>Q140 CSF vs Q140 GM1	$p=0.0007$<br>$p=0.0232$
Figure 5	B	Q7/7 CSF vs Q140 CSF Males<br>Q140 CSF vs Q140 GM1 Males	$p=0.0007$<br>$p=0.0007$
	C	Q7/7 CSF vs Q140 CSF<br>Q140 CSF vs Q140 GM1	$p=0.014$<br>$p=0.007$
	E	Q7/7 CSF vs Q140 CSF Hind<br>Q140 CSF vs Q140 GM1 Fore	$p=0.002$<br>$p=0.013$
	F	Q7/7 CSF vs Q140 CSF LF/LH<br>Q140 CSF vs Q140 GM1 LF/LH<br>Q7/7 CSF vs Q140 GM1 RH/LH	$p<0.0001$<br>$p<0.0001$<br>$p=0.013$
Figure 6	A	WT CSF vs YAC128 CSF closed<br>YAC128 CSF vs GM1 closed<br>YAC 128 CSF vs GM1 open	$p=0.0157$<br>$p=0.0009$<br>$p=0.0004$
	B	WT CSF vs YAC128 CSF<br>YAC128 CSF vs GM1	$p=0.0028$<br>$p=0.0306$
	C	Q7/7 CSF vs Q140 CSF dark<br>Q140 CSF vs Q140 GM1 dark<br>Q7/7 CSF vs Q140 CSF light<br>Q140 CSF vs Q140 GM1 light<br>Q7/7 CSF vs Q140 CSF entries<br>Q140 CSF vs Q140 GM1 entries	$p=0.0177$<br>$p=0.0049$<br>$p=0.0011$<br>$p=0.0218$<br>$p=0.0016$<br>$p=0.015$

Appendix Table S1 – *P* values for pairwise comparisons reported in the manuscript (Cont.)

Figure	Panel	Groups compared	*p*-value
Figure 6	D	WT CSF vs YAC128 CSF<br>YAC128 CSF vs YAC128 GM1<br>Q7/7 CSF vs Q140 CSF<br>Q140 CSF vs Q140 GM1<br>WT CSF vs R6/2 CSF<br>R6/2 CSF vs R6/2 GM1	p=0.0079<br>p=0.0004<br>p<0.0001<br>p=0.0007<br>p=0.0054<br>p=0.0336
	E	WT CSF vs YAC128 CSF immobile<br>YAC128 CSF vs YAC128 GM1 immobile<br>WT CSF vs YAC128 CSF swimming<br>YAC128 CSF vs YAC128 GM1 swimming	p<0.0001<br>p=0.0015<br>p=0.0003<br>p=0.0024
	F	WT CSF vs YAC128 CSF<br>YAC128 CSF vs YAC128 GM1	p=0.0024<br>p=0.0306
Figure 7	A	WT CSF vs YAC128 CSF<br>YAC128 CSF vs YAC128 GM1	p=0.0342<br>p=0.0136
	B	WT CSF vs YAC128 CSF<br>YAC128 CSF vs YAC128 GM1	p=0.0448<br>p=0.0129
	C	Q7/7 CSF vs Q140 CSF<br>Q140 CSF vs Q140 GM1	p=0.0053<br>p=0.0418
	D	Q7/7 CSF vs Q7/140 CSF<br>Q7/140 CSF vs Q140/140 CSF<br>Q7/140 CSF vs Q7/140 GM1	p=0.0011<br>p=0.0187<br>p=0.0002
	E	Q7/7 CSF vs Q140 CSF<br>Q140 CSF vs Q140 GM1	p=0.0055<br>p=0.0055
Figure 8	Glutamate	WT CSF vs YAC128 CSF<br>YAC128 CSF vs YAC128 GM1	p=0.0091<br>p=0.0399
	GABA	WT CSF vs YAC128 CSF<br>YAC128 CSF vs YAC128 GM1	p=0.036<br>p=0.0099
	Glycine	Main effect of genotype	p=0.0132
	L-Serine	YAC128 CSF vs YAC128 GM1	p=0.0151
	D-Serine	YAC128 CSF vs YAC128 GM1	p=0.0234
Figure EV1	pDARRP32	Q7/7 CSF vs Q7/140 CSF<br>Q7/140 CSF vs Q7/140 GM1<br>Q7/140 GM1 vs Q140/140 CSF<br>Q7/140 GM1 vs Q140/140 GM1	p<0.0001<br>p=0.0013<br>p=0.0012<br>p<0.0001
	DARRP32	Q7/7 CSF vs Q7/140 CSF<br>Q7/140 CSF vs Q7/140 GM1<br>Q7/140 GM1 vs Q140/140 CSF<br>Q7/140 GM1 vs Q140/140 GM1	p<0.0001<br>p=0.0146<br>p=0.0006<br>p<0.0001

Appendix Table S1 – *P* values for pairwise comparisons reported in the manuscript (Cont.)

Figure	Panel	Groups compared	*p*-value
Figure EV2	A	WT CSF vs YAC128 CSF swimming<br>YAC128 CSF vs YAC128 GM1 swimming<br>WT CSF vs YAC128 CSF immobile<br>YAC128 CSF vs YAC128 GM1 immobile<br>WT CSF vs WT GM1 immobile	*p*=0.0341<br>*p*=0.0026<br>*p*=0.0396<br>*p*=0.0006<br>*p*=0.0172
Figure EV2	B	Q7/7 CSF vs Q140 GM1 immobile<br>Q140 CSF vs Q140 GM1 immobile<br>Q140 CSF vs Q140 GM1 swimming	*p*=0.0072<br>*p*=0.0162<br>*p*=0.0079
Appendix Figure S4	B	YAC128 Saline vs YAC128 adinazolam<br>YAC128 Saline vs YAC128 adinazolam	*p*=0.0165<br>*p*=0.005
Appendix Figure S5	C	YAC128 Saline vs YAC128 imipramine<br>YAC128 Saline vs YAC128 imipramine	*p*<0.0001<br>*p*<0.0001

Appendix Table S2 – Statistical analysis of behavioural differences between Q7/140 and Q140/140 mice

Phenotype /Test	Q7/140 *vs* Q140/140	Main effect of genotype (Two-way ANOVA)	Main effect of interaction (Two-way ANOVA)
DARPP32 expression	≠	$F(1,46)=1.890$ $p=0.0021$	$F(1,46)=10.61$, $p=0.1759$
pDARPP32 expression	≠	$F(1,47)=27.64$, $p<0.0001$	$F(1,47)=28.24$ $p<0.0001$
mHTT expression Cx	=	$F(1,11)=0.37$, $p=0.5573$	$F(1,11)=0.30$, $p=0.5945$
mHTT expression St	=	$F(1,11)=0.015$, $p=0.86$	$F(1,11)=0.033$ $p=0.9033$
Horizontal ladder	=	$F(1,31)=1.98$, $p=0.1696$	$F(1,31)=0.26$ $p=0.6114$
Open field	=	$F(1,48)=0.94$, $p=0.3378$	$F(1,48)=0.0036$ $p=0.9521$
Climbing test	=	$F(1,53)=0.14$, $p=0.7097$	$F(1,53)=0.025$ $p=0.8758$
Fixed rotarod	=	$F(1,23)=0.0024$, $p=0.964$	$F(1,23)=0.35$ $p=0.5581$
Time in dark box	=	$F(1,49)=0.6085$, $p=0.4391$	$F(1,49)$ $=0.0082$, $p=0.9282$
Time in light box	=	$F(1,51)=3.680$, $p=0.0607$	$F(1,51)=0.01927$, $p=0.89$
Light-dark box – total entries	=	$F(1,52)=0.0028$, $p=0.8686$	$F(1,52)=0.030$, $p=0.8627$
Time immobile	=	$F(1,35)=1.40$, $p=0.2446$	$F(1,35)=0.65$, $p=0.4273$
Time swimming	=	$F(1,36)=0.18$, $p=0.6735$	$F(1,36)=0.042$, $p=0.60$
Time climbing	=	$F(1,37)=0.32$, $p=0.5746$	$F(1,37)=0.28$, $p=0.5997$
Intersession habituation	=	$F(1,44)=0.39$ $p=0.5357$	$F(1,44)=0.20$, $p=0.6539$
Intrasession habituation	=	$F(1,45)=0.14$, $p=0.7138$	$F(1,45)=0.65$, $p=0.4244$
Y-maze correct alternations	≠	$F(1,37)=5.28$, $p=0.0274$	$F(1,37)=1.57$, $p=0.2175$

Two –way ANOVA was used to compare values obtained for heterozygous Q7/Q140 and homozygous Q140/140 mice in the various analyses and tests indicated in the left column. The symbol "=" indicates that values obtained for the two genotypes were similar and data were combined in the corresponding graphs and figures; "≠" indicates that values for the two genotypes were different and were analysed separately.

A

Animal Model	Age at the start of Treatment	Duration of Treatment	Tests and Analyses Performed
**R6/2**	6 weeks (cohort 1)	28 days	• **Neuropathology**
**R6/2**	8 weeks (cohort 2)	28 days	• **Motor behaviour:** open-field, ladder<br>• **Non-motor behaviour:** spontaneous defecation<br>• **Biochemistry:** Insoluble HTT
**Q140**	6.4 ± 0.86 months	42 days	• **Motor behaviour:** ladder, open field, climbing, rotarod, gait analysis<br>• **Non-motor behaviour:** forced swim test, light-dark box, spontaneous defecation, open field habituation, Y-maze<br>• **Biochemistry:** Soluble and insoluble HTT, DARPP32
**YAC128**	6.4 ± 0.35 months (cohort 1)	28 days	• **Non-motor behaviour:** forced swim test, elevated plus maze, open field habituation, three chamber test<br>• **Neurochemistry:** amino acids and biogenic amines
**YAC128**	9.2 ± 0.37 months (cohort 2)	28 days	• **Non-motor behaviour:** forced swim test, novelty-induced hypophagia, spontaneous defecation, open field habituation, nesting

B

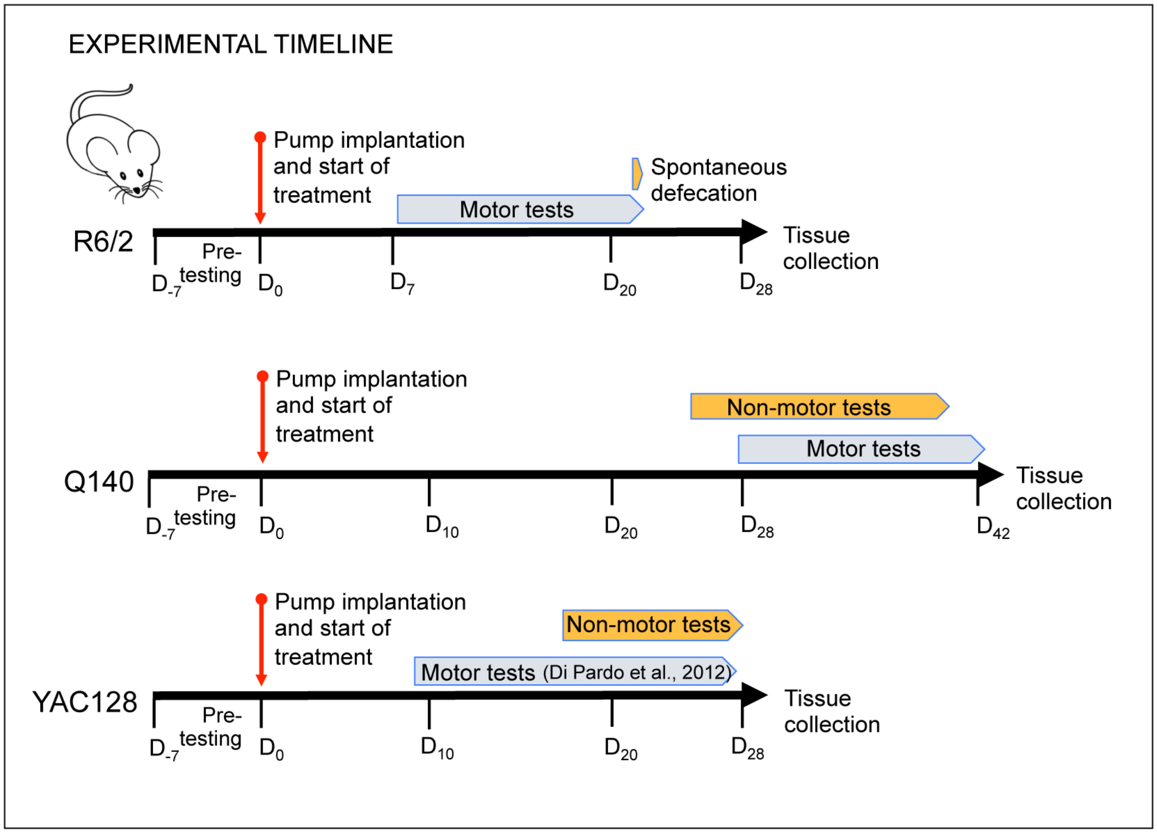

Appendix Fig. S1. Animal models and experimental design. (A) Age, duration of treatment, tests and analyses performed for each animal model used in this study. **(B)** Timeline of treatment and testing for each animal model used in this study. For each specific test, all experimental groups were tested on the same day after the beginning of treatment.

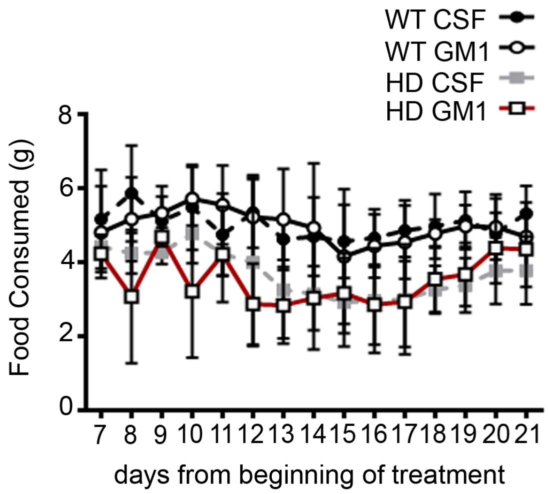

Appendix Fig. S2. Food consumption is not affected by GM1 in R6/2 mice. R6/2 mice were treated with cerebro-spinal fluid (CSF, vehicle) or GM1. Average mouse food consumption from day 7 to 21 of treatment is reported. N = 16 WT CSF, 14 WT GM1, 8 R6/2 CSF, 7 R6/2 GM1. Differences among groups were not statistically significant. Repeated measures two-way ANOVA with Bonferroni post-test.

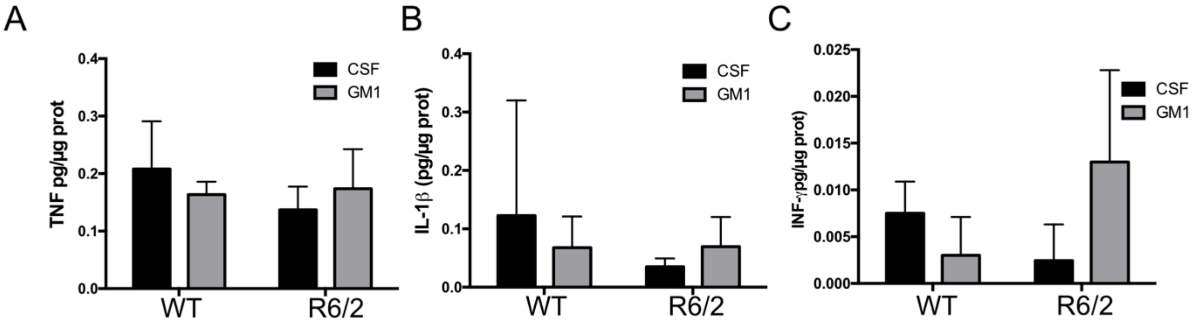

Appendix Fig. S3. Expression of major inflammatory cytokines is comparable between WT and R6/2 mice and is not significantly affected by treatment with GM1. Cortical levels of TNF **(A)**, IL-1β **(B)** and INFγ **(C)**, were measure in WT and R6/2 mice treated for 28 days with CSF or GM1. N= 5 WT CSF, 5 WT GM1, 4 R6/2 CSF, 7 R6/2 GM1. Bars represent mean values ± SD. Differences were not statistically significant. Two-way ANOVA with Holm-Sidak's multiple comparisons test.

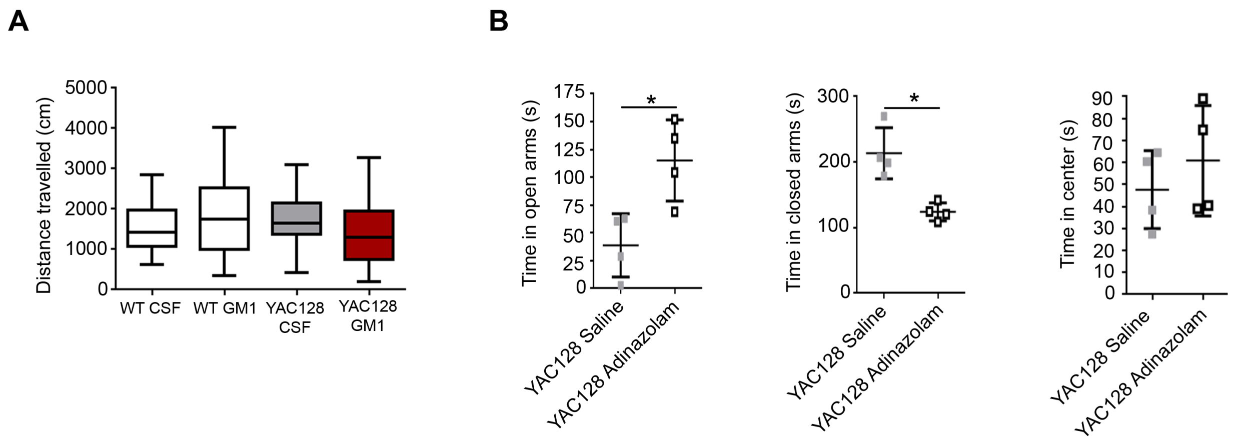

Appendix Fig. S4. The behaviour of YAC128 mice in the elevated plus maze does not depend on mouse motor function and is affected by acute adinazolam treatment.

(A) The distance travelled by mice in an open field arena during a 5 min session was measured. Box-and-whisker plots show median, maximum and minimum values. N = 15 WT CSF, 14 WT GM1, 13 YAC128 CSF, 19 YAC128 GM1. Two-way ANOVA with Bonferroni post-test.

(B) Thirty minutes prior to placement in the elevated plus maze, YAC128 mice were injected intraperitoneally with saline or 2.5 mg/kg adinazolam. Time spent in each of the three compartments of the elevated plus maze was recorded. Individual values, as well as means and SD are shown. N = 4 YAC128 saline, 4 YAC128 adinazolam. Two-tailed Student's *t*-test. *$p<0.05$

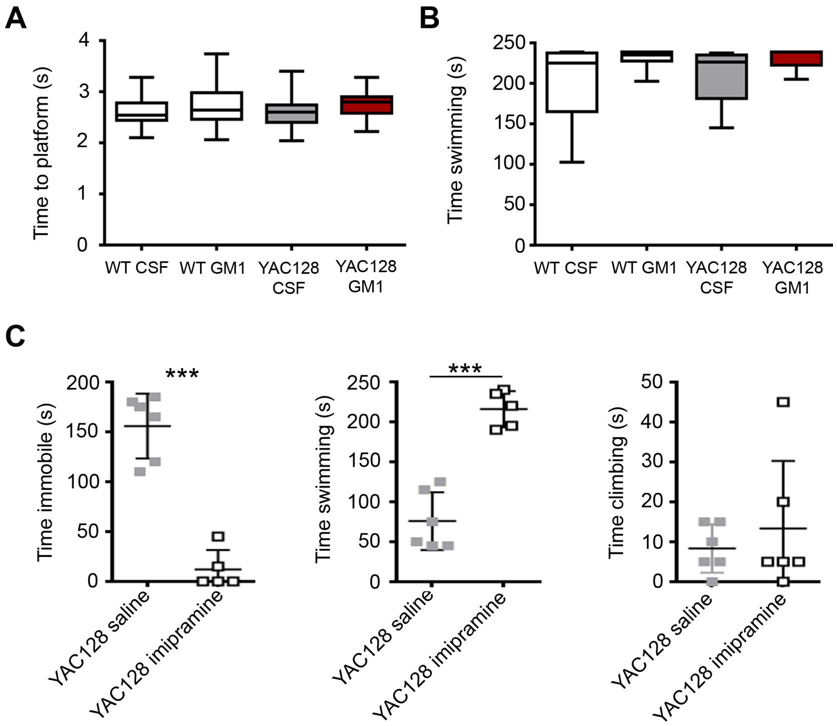

Appendix Fig. S5. The behaviour of YAC128 mice in the forced swim test does not depend on mouse motor functions and is corrected by the anti-depressant imipramine.

(A) YAC128 mice and WT littermates were trained to swim to a platform placed at one end of a narrow rectangular water pool. The time required to swim to the platform was recorded. **(B)** Swimming endurance of YAC128 and WT mice in a circular water pool was measured during a 6 min-session (same duration as the forced swim test). As in the forced swim test, time mice spent swimming or floating was measured in the last 4 minutes of the test. N = 21 WT CSF, 23 WT GM1, 17 YAC128 CSF, 16 YAC128 GM1. Box-and-whisker plots show median, maximum and minimum values. Two-way ANOVA with Bonferroni post-test. **(C)** Thirty min prior to placement in the forced swim test apparatus, 6-10 month-old YAC128 mice were administered saline or 10 mg/kg imipramine by intraperitoneal injection. The forced swim test was run for a total of 6 min and time spent swimming, floating or climbing was recorded in the last 4 minutes. N = 6 YAC128 saline, 6 YAC128 imipramine. Two-tailed Student's *t*-test. ***p<0.001.

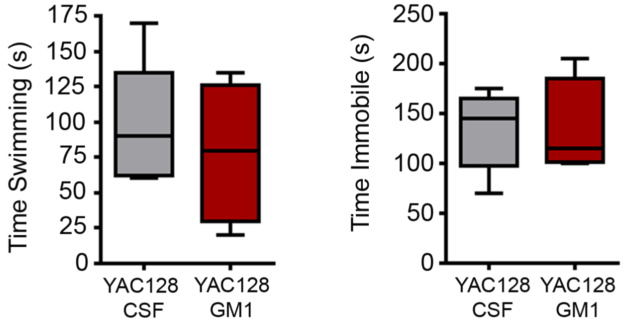

Appendix Fig. S6. Effect of short-term treatment with GM1 on the performance of YAC128 mice in the forced swim test.

Six month-old YAC128 mice treated with vehicle (CSF) or GM1 were assessed in the forced swim test 10 days after the beginning of treatment. Short-term GM1 treatment did not improve the performance of YAC128 mice. N = 5 YAC128 CSF and 4 YAC128 GM1. Box-and-whisker plots show median, maximum and minimum values. Two-tailed Student *t*-test.
