# Supplementary material for: Disease‐modifying effects of ganglioside GM1 in Huntington's disease models
Source: EMBO Mol Med. 2017 Oct 9;9(11):1537–57. doi: 10.15252/emmm.201707763 (PMC5666311; doi:10.15252/emmm.201707763)
Supplement: Supplementary file 2 — Expanded View Figures PDF [file EMMM-9-1537-s002.pdf]

Expanded View Figures

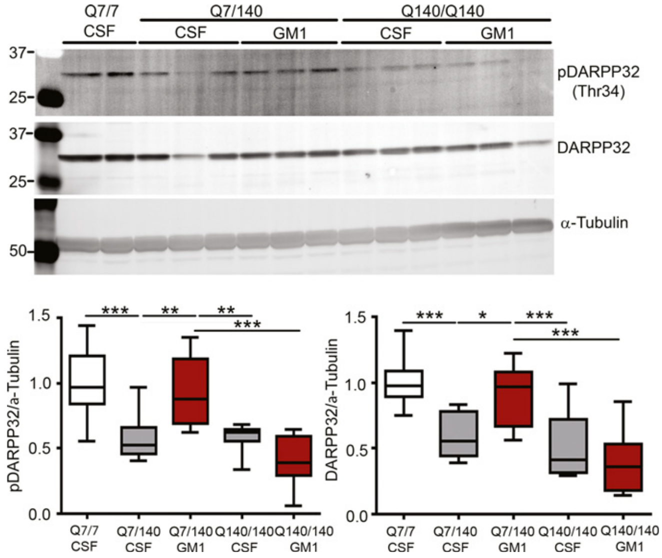

Figure EV1. GM1 increases DARPP32 and its phosphorylation in Q7/Q140 mice.

Representative immunoblots and densitometric analysis of DARPP32 expression and phospo-Thr34 DARPP32 (p-DARPP32) after treatment with GM1. *N* = 20 Q7/7 CSF, 13 Q7/140 CSF, 9 Q7/140 GM1, 14 Q140/140 CSF, 15 Q140/140 GM1. Box-and-whisker plots show median, maximum and minimum values. One-way ANOVA with Tukey's post-test. $^*P < 0.05$, $^{**}P < 0.01$, $^{***}P < 0.001$.

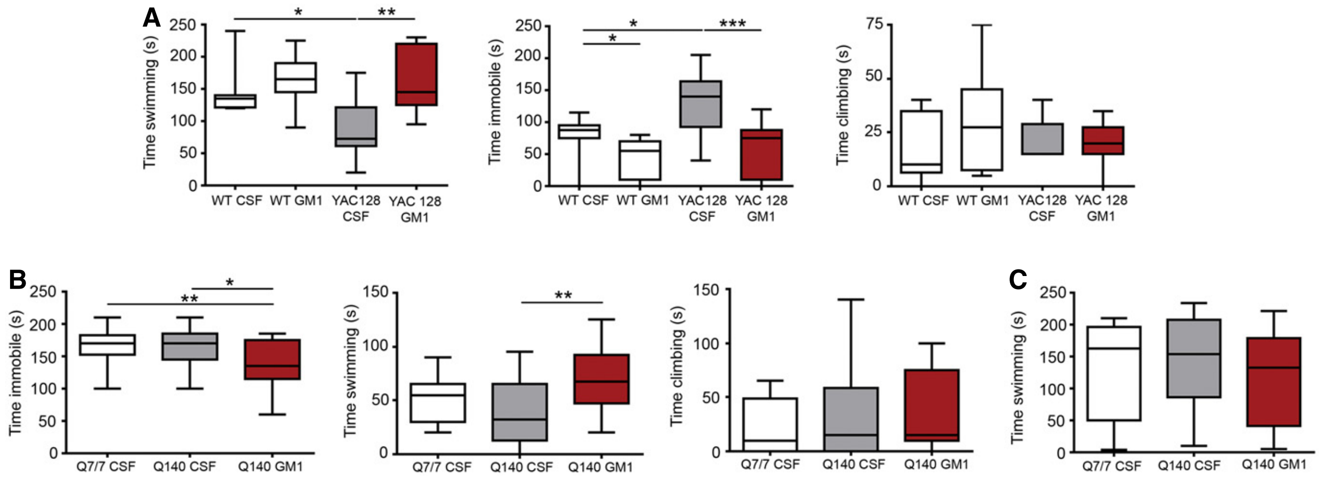

Figure EV2. GM1 decreases depression-like behaviour in Q140 mice and in older WT mice.

A The behaviour of 9-month-old YAC128 mice and WT littermates was assessed in the forced swim test during treatment with cerebro-spinal fluid (CSF, vehicle) or GM1. *N* = 12 WT CSF, 12 WT GM1, 8 YAC128 CSF, 9 YAC128 GM1.

B,C Q140 mice and Q7/7 littermates were tested in the forced swim test (B) as well as in the open pool test (C) to measure swimming endurance. *N* = 20 Q7/7 CSF, 20 Q140 CSF, 19 Q140 GM1.

Data information: Box-and-whisker plots show median, maximum and minimum values. Two-way (A) or one-way ANOVA (B, C) with Bonferroni post-test. $^*P < 0.05$, $^{**}P < 0.01$, $^{***}P < 0.001$.

 © 2017 The Authors
