# Supplementary material for: Disease‐modifying effects of ganglioside GM1 in Huntington's disease models
Source: EMBO Mol Med. 2017 Oct 9;9(11):1537–57. doi: 10.15252/emmm.201707763 (PMC5666311; doi:10.15252/emmm.201707763)

A **Fig. 2C and 2F**. Original grey scale images of Li-Cor scan (700 channel), **overexposed**, showing the position where the PVDF membrane was cut immediately after transfer, between the 37 and 25 kDa weight marker (red arrow). The top part of the membrane was subsequently probed with anti-GFAP and/or anti-tubulin antibodies; the bottom part was probed with anti-IbaI antibodies (see images on the right).

B **Fig. 2C and 2F**. Original Li-Cor color scans showing the overlay of the 700 and 800 channels for each portion of the membrane. Top and bottom panels correspond to top and bottom panels in A, respectively, and were probed with anti-tubulin and/or anti-GFAP antibodies (top) or with anti Iba-1 antibodies (bottom).

C Grey scale images of 700 channel scan from which the images in Fig. 2C -Cortex (top panel, boxed) and Fig. 2F - Cortex (bottom panel, boxed) were derived.

D Grey scale image of the 800 channel scan from which the image in Fig. 2C and 2F - Cortex was derived (boxed).

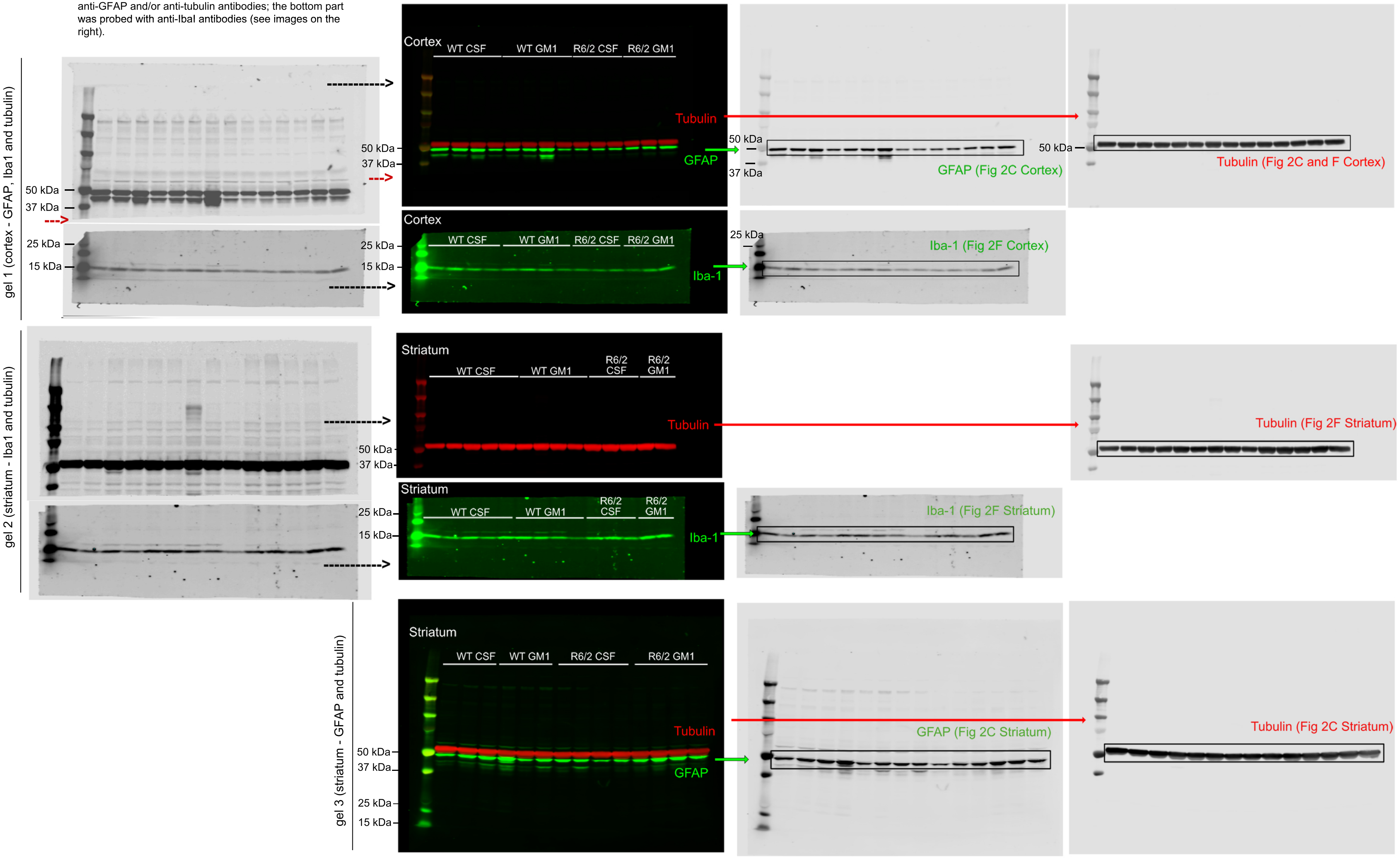

Supplement: Supplementary file 4 — Source Data for Figure 2 [file EMMM-9-1537-s003.pdf]
